# DeepFaune New England: A Species Classification Model for Trail Camera Images in Northeastern North America

**DOI:** 10.1002/ece3.72174

**Published:** 2025-11-14

**Authors:** Laurence A. Clarfeld, Katherina D. Gieder, Angela Fuller, Zhongqi Miao, Alexej P. K. Sirén, Shevenell M. Webb, Toni Lyn Morelli, Tammy L. Wilson, Jillian Kilborn, Catherine B. Callahan, Leighlan S. Prout, Rachel Cliche, Riley K. Patry, Christopher Bernier, Susan Staats, Scott Wixsom, Therese M. Donovan

**Affiliations:** ^1^ Vermont Cooperative Fish and Wildlife Research Unit, 302 Aiken Center University of Vermont Burlington Vermont USA; ^2^ Vermont Department of Fish and Wildlife Rutland Vermont USA; ^3^ U.S. Geological Survey, New York Cooperative Fish and Wildlife Research Unit, Department of Natural Resources and the Environment Cornell University Ithaca New York USA; ^4^ AI for Good Research Lab Microsoft Seattle Washington USA; ^5^ Earth Systems Research Center, Institute for the Study of Earth, Oceans, and Space University of New Hampshire Durham New Hampshire USA; ^6^ Maine Department of Inland Fisheries and Wildlife Augusta Maine USA; ^7^ U.S. Geological Survey Northeast Climate Adaptation Science Center Amherst Massachusetts USA; ^8^ U.S. Geological Survey, Massachusetts Cooperative Fish and Wildlife Research Unit, Department of Environmental Conservation University of Massachusetts Amherst Massachusetts USA; ^9^ New Hampshire Fish & Game Department Concord New Hampshire USA; ^10^ New Hampshire Department of Information Technology Concord New Hampshire USA; ^11^ U.S. Forest Service White Mountain National Forest Campton New Hampshire USA; ^12^ Silvio O Conte National Fish and Wildlife Refuge Brunswick Vermont USA; ^13^ Dartmouth College Woodlands Milan New Hampshire USA; ^14^ Vermont Department of Fish and Wildlife Springfield Vermont USA; ^15^ Green Mountain and Finger Lakes National Forests Rochester Vermont USA; ^16^ Green Mountain National Forest Manchester Center Vermont USA; ^17^ U.S. Geological Survey, Vermont Cooperative Fish and Wildlife Research Unit, 302 Aiken Center University of Vermont Burlington Vermont USA

**Keywords:** camera trap, image classification, machine learning, species distribution modeling, trail camera, wildlife monitoring

## Abstract

The DeepFaune New England model classifies wildlife species in trail camera images, identifying 24 taxa from northeastern North America with high (97%) accuracy. The model was adapted from the DeepFaune model for identifying European wildlife, demonstrating the practicality of transfer learning across continents. The majority of training data is openly licensed, and the model itself is open source, enabling easy integration into camera trapping workflows. The open source software is available at (https://code.usgs.gov/vtcfwru/deepfaune‐new‐england), and has been further integrated into the PyTorch‐Wildlife framework.

## Graphical abstract

The DeepFaune New England model classifies wildlife species in trail camera images, identifying 24 taxa from northeastern North America with high (97%) accuracy. The model was adapted from the DeepFaune model for identifying European wildlife, demonstrating the practicality of transfer learning across continents. The majority of training data is openly licensed, and the model itself is openly sourced, enabling easy integration into camera trapping workflows.

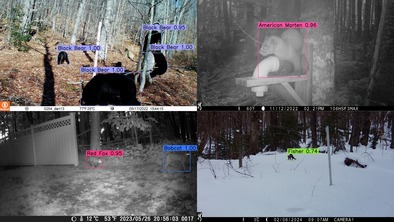

## 1 Introduction

Monitoring wildlife via trail cameras has exploded in popularity in recent years, spurred by technological advances in hardware, data storage, and processing that have made collecting data, particularly over large spatial and temporal scales, more cost‐effective than ever before (Steenweg et al. 2017). Trail cameras offer a standardized method for monitoring across large spatial and temporal scales, making them an ideal methodology for measuring shifts in distribution and abundance as wildlife respond to environmental change (Steenweg et al. 2017). As the volume of data collected increases, labeling images for the presence of target wildlife species can become a bottleneck that impedes the timely analysis of trail camera data, potentially delaying conservation and management decision‐making. To mitigate this, researchers are increasingly turning to machine learning (ML) to automate species detection and identification.

Numerous open source models are available to researchers who seek to use ML to identify animals from trail camera imagery. Species classification models have been trained on camera trapping datasets from Africa (Villa et al. 2017; Norouzzadeh et al. 2018), Europe (Choiński et al. 2021; Rigoudy et al. 2023; Schneider et al. 2023), Asia (Wang et al. 2022), Latin America (Hernandez et al. 2024), North America (Tabak et al. 2020, 2022), and globally (Ahumada et al. 2020). However, few, if any, of these models are trained on data with strong representation from northeastern North America, including New England. This coverage gap presents a challenge for researchers from this region who must either use existing models that may be prone to misclassification, train their own models, or manually annotate images without the assistance of ML. Subsequently, there is a need for an open source regional model specialized on northeastern taxa. To fill this gap, we trained DeepFaune New England, a computer vision model for classifying the common taxa from northeastern North America that appear in trail camera imagery (Clarfeld, Tracey, et al. 2025).

To train our model, we used transfer learning, a technique whereby existing models are re‐trained on new data (Tan et al. 2018). The base model that we re‐trained was DeepFaune, a self‐supervised vision transformer (ViT) model for classifying European wildlife from trail camera images (Rigoudy et al. 2023). DeepFaune relies on bounding boxes that localize animals within an image. These bounding boxes can be manually annotated or inferred by an object detection model such as MegaDetector (Beery et al. 2019). We retrained DeepFaune with a dataset of trail camera images representing 24 species and higher‐level taxa (e.g., family‐level tags) from New England, including a “no‐species” label representing the absence of animals. The resulting model, which we call DeepFaune New England (DFNE), achieves 97% accuracy when evaluated on unseen, out‐of‐sample data. Our objectives herein are to describe: (1) the formation of our dataset; (2) our training and evaluation methodology; and (3) how to use the model.

## 2 Dataset Formation

We combined data from over a dozen camera trapping projects, most of which were publicly available, and approximately half of which were collected in the northeastern states of Maine, Massachusetts, New Hampshire, New York, and Vermont (Table 1). Metadata for all projects were stored in project‐specific SQLite databases created with the R package *AMMonitor* (Clarfeld et al. 2024; Clarfeld, Tang, et al. 2025), providing a unifying framework to facilitate data integration. All images were analyzed using MegaDetector v.5a (Beery et al. 2019) to localize humans and animals within images and were independently labeled by people trained and qualified to identify the species or higher‐order taxon present in the image. The bounding boxes and associated confidence scores of MegaDetector detections were stored in each database along with the human‐generated taxon labels. MegaDetector does not classify the taxon of animals in images, but we used the human‐produced labels to infer which taxon was within each bounding box.

**TABLE 1:** Data sources for training DFNE, including dataset names, repositories, citations, and total number of photos and taxa.

Dataset	Citation	Photo count	Taxon count
Caltech Camera Traps ^1^	Beery et al. (2018)	36,751	12
Dartmouth College Woodlands Wildlife Monitoring Project ^2^	Patry et al. (2024)	434	15
ENA24‐detection ^1^	Yousif et al. (2019)	4771	14
iNaturalist ^3^	iNaturalist (2024)	10,681	13
Maine Department of Inland Fisheries and Wildlife Furbearer Project ^2^	Webb et al. (2024)	27,771	19
Maine Department of Inland Fisheries and Wildlife Moose Project ^2^	Kantar et al. (2024)	13,403	19
Massachusetts Wildlife Monitoring Project ^2^	Wilson et al. (2024)	15,714	22
New Hampshire Fish and Game Department ^2^	Jones et al. (2024)	7498	21
New York Cooperative Fish & Wildlife Research Unit/New York State Department of Environmental Conservation ^4^	Twining et al. (2024)	45,272	16
North American Camera Trap Images ^1^	Tabak et al. (2019)	33,529	10
Silvio O Conte National Fish and Wildlife Refuge Wildlife Monitoring Project ^2^	Cliche et al. (2024)	4014	17
SiMPL Wildlife Magnet Project ^2^	Morelli et al. (2024)	18,577	13
USDA (U.S. Department of Agriculture) Green Mountain National Forest ^2^	Gieder, Bernier, Staats, et al. (2024)	12,016	19
USDA White Mountain National Forest ^2^	Prout et al. (2024)	7384	18
Vermont Fish and Wildlife Department ^2^	Gieder, Bernier, Royar, et al. (2024)	9733	18

*Note:* The dataset repository is indicated by superscripts next to the Dataset name: (1) LILA (Labeled Information Library of Alexandria); (2) ScienceBase; (3) iNaturalist; and (4) CFWRU (Cooperative Fish and Wildlife Research Unit).

Candidate training samples were generated by cropping the original trail camera image using the bounding box produced by MegaDetector. We discarded detections where either (1) the image had been labeled as containing an animal that wasn't on our list of target taxa; (2) the detection confidence score was below 0.75 (for images with animals); or (3) the image contained more than one species of animal. Images with the “no‐species” label were generated from any images labeled by human observers as not having an animal, but where MegaDetector predicted an animal. These were included to train DFNE to detect false positive predictions of animals by an object detection model (such as MegaDetector).

Due to variation in species abundance, the candidate training dataset was highly imbalanced and non‐uniformly distributed across species, projects, and locations. To address this, we applied importance sampling to partition the data into training (80%), validation (10%), and testing (10%) sets while maintaining balance and independence. Specifically, sampling ensured that for each set, no taxon had more than 12,000 images, image locations were mutually exclusive between subsets, and the relative frequency of taxa was approximately equal within each subset. We excluded taxa for which we had fewer than 3000 candidate training samples. The final partitioned training/testing/validation datasets contained 247,548 images representing 24 taxa, including a “no‐species” label (Table 2). The taxa included in the training dataset covered nearly 99% of all available images from the northeastern United States. Each taxon typically had 10–12,000 samples in the final dataset, but the most under‐represented taxon (Mouse sp.) had just 3543 examples. More information about the origins of our training data is available in the references from Table 1 and additional metadata for our training set, including additional details on data collection protocols, are available on USGS ScienceBase (Clarfeld, Gieder, et al. 2025).

**TABLE 2:** Taxa covered by DFNE V1.0.0 including common name, scientific name, taxonomic rank, and taxonomic serial number (TSN), as defined by the Integrated Taxonomic Information System (https://www.itis.gov/).

Common name	Scientific name	Rank	TSN
American Marten	*Martes americana*	Species	180559
Bird sp.	*Aves*	Class	174371
Black bear	*Ursus americanus*	Species	180544
Bobcat	*Lynx rufus*	Species	180582
Coyote	*Canis latrans*	Species	180599
Domestic cat	*Felis catus*	Species	183798
Domestic cow	*Bos taurus*	Species	183838
Domestic dog	*Canis lupus familiaris*	Subspecies	726821
Fisher	*Pekania pennanti*	Species	1086061
Gray fox	*Urocyon cinereoargenteus*	Species	180609
Gray squirrel	*Sciurus carolinensis*	Species	180175
Human	*Homo sapiens*	Species	180092
Moose	*Alces americanus*	Species	898420
Mouse sp.	*Rodentia*	Order	180130
No‐species			N/A
Opossum	*Didelphis virginiana*	Species	179921
Raccoon	*Procyon lotor*	Species	180575
Red fox	*Vulpes vulpes*	Species	180604
Red squirrel	*Tamiasciurus hudsonicus*	Species	180166
Skunk	*Mephitis mephitis*	Species	180562
Snowshoe hare	*Lepus americanus*	Species	180112
White‐tailed deer	*Odocoileus virginianus*	Species	180699
Wild boar	*Sus scrofa*	Species	180722
Wild turkey	*Meleagris gallopavo*	Species	176136

## 3 Model Training and Evaluation

**FIGURE 1:**
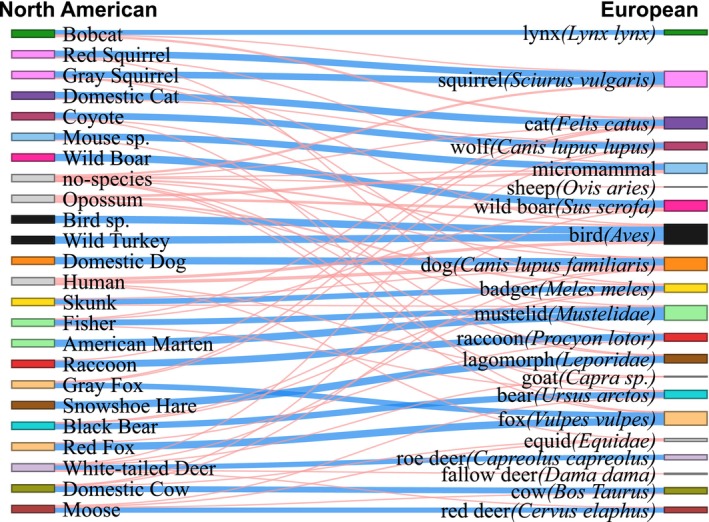
Sankey diagram showing mapping between North American and European taxa. We consider “matched” taxa as those with > 50% of samples corresponding to the same match. Pipes between the left and right sides are blue where they match and red where they do not match. The colored boxes of corresponding North American and European taxa are matched; however, gray boxes indicate taxa where there was no clear mapping between North American and European taxa. The thickness of each pipe represents the relative abundance of matching samples.

The DeepFaune model (Rigoudy et al. 2023), based on the DINOv2 ViT model architecture (Darcet et al. 2023; Oquab et al. 2023), classifies 30 European taxa with > 95% accuracy and was used as our base model. To initialize the model before re‐training it, we used DeepFaune to classify 20 random samples of each North American taxon from our training set (Table 2) to form a mapping between North American and European taxa. This resulted in a majority (> 50% match) mapping for 21 of our 24 classes (Figure 1). Thirteen North American taxa had 1‐to‐1 mappings with European taxa (e.g., White‐tailed Deer ( *Odocoileus virginianus* ) mapped to western roe deer ( *Capreolus capreolus* ) and skunk ( *Mephitis mephitis* ) mapped to European badger ( *Meles meles* )). Four pairs of North American species mapped 2‐to‐1 to European species (e.g., American Marten ( *Martes americana* ) and Fisher ( *Pekania pennanti* ) both mapped to mustelid (*Mustelidae*)). Three North American taxa (“no‐species,” Human and Opossum ( *Didelphis virginiana* )) did not map to any European taxa included in the original DeepFaune class list.

We modified DeepFaune's final, fully connected layer to contain 24 output nodes (one for each North American taxon) and initialized the weights for each with weights from the most similar European taxon from the original model. For taxa with no mapping, we initialized weights using the Xavier technique by sampling them from a uniform distribution of ($-\frac{1}{n},\frac{1}{n}$) where n is the number of classes (Glorot and Bengio 2010). All weights were then perturbed with a random jitter sampled from a normal distribution with mean of 0 and standard deviation equal to 1% of the initial weights to promote better exploration of the solution space and avoid early convergence at local minima.

**FIGURE 2:**
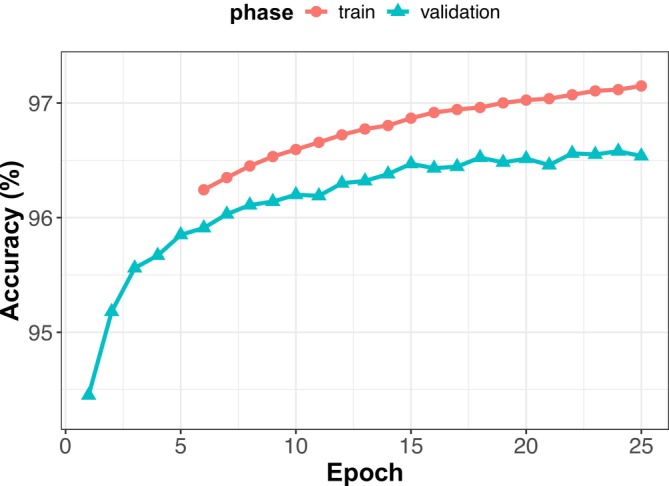
Learning curve showing the model accuracy after each epoch of training when evaluated on the training and validation sets (note, training accuracy is missing for the first 5 epochs). Note, the y‐axis ranges from 94.3% to 97.3%.

We retrained the fully connected layer using a stochastic gradient descent optimizer with a learning rate of 1e−4, a batch size of 16, and a weighted cross‐entropy loss function. The model weights from the best‐performing epoch (on the validation set) were selected, and the final evaluation was performed on the testing set. The model was trained on the Tallgrass high‐performance computing cluster (Falgout et al. 2025) using Pytorch (Ansel et al. 2024), a deep learning framework for Python (Python Software Foundation 2021). We trained the model for 25 epochs, at which point performance improvement in the validation set began to plateau (Figure 2). After a single training epoch, validation accuracy had already surpassed 94%. The highest validation accuracy was achieved in the 24th epoch. We used the weights from this epoch for our final model and evaluated performance on the testing set. The final model, named DeepFaune New England, was 97% accurate, with most classes surpassing 95% accuracy (Table 3). Performance metrics included precision (the proportion of predicted instances of a taxon that are correct) and recall (from all observations of a taxon, the proportion that are correctly predicted). Balanced Accuracy is the average of recall (sensitivity) and specificity, reflecting the model's ability to both detect a given taxon and to avoid misclassifying other taxa as that taxon. Precision and recall were consistently high and well‐balanced across all classes. Only two taxa had precision/recall below 0.9: the “no‐species” class had a precision of 0.88 and Mouse sp. had a recall of 0.89. Balanced accuracy exceeded 95% for all taxa.

**TABLE 3:** Performance metrics of the final model on the testing set for each taxon.

Taxon	Precision	Recall	Balanced accuracy
American Marten	0.98	0.99	1.00
Bird sp.	0.96	0.99	0.99
Black bear	0.97	0.98	0.99
Bobcat	0.97	0.95	0.97
Coyote	0.98	0.96	0.98
Domestic cat	0.98	0.97	0.98
Domestic cow	0.99	0.96	0.98
Domestic dog	0.96	0.97	0.98
Fisher	0.98	0.96	0.98
Gray fox	0.95	0.99	0.99
Gray squirrel	0.98	0.97	0.99
Human	0.98	0.98	0.99
Moose	0.94	0.97	0.99
Mouse sp.	0.98	0.89	0.95
No‐species	0.88	0.92	0.96
Opossum	0.99	0.99	0.99
Raccoon	0.94	0.96	0.98
Red fox	0.96	0.95	0.98
Red squirrel	0.99	0.96	0.98
Skunk	0.97	0.98	0.99
Snowshoe hare	0.97	0.98	0.99
White‐tailed deer	0.96	0.95	0.98
Wild boar	0.99	0.99	0.99
Wild turkey	1.00	0.99	0.99

*Note:* Metrics include precision, recall, and balanced accuracy.

We compared the performance of DFNE to SpeciesNet, a Python package released in 2025 that provides an ensemble of models for classifying wildlife in trail camera imagery (Gadot et al. 2024). Details of this comparison are included in Appendix.

## 4 Using DeepFaune New England

### 4.1 Installation and Setup

The canonical home of the DFNE model is (https://code.usgs.gov/vtcfwru/deepfaune‐new‐england), where issues and merge requests can be submitted. The code repository contains scripts, sample images, and fully worked examples, and it details three steps for setting up DFNE with the minimal number of dependencies. First, users clone the repository to download the model code. Next, users download the model weights from USGS ScienceBase (Clarfeld, Gieder, et al. 2025) to the repository's “models” folder. Finally, users install software dependencies (e.g., a Python Distribution, such as Miniconda (Anaconda Inc 2025)).

Once the dependencies are installed and loaded, create the model using:classifier_model = model (weights = "models/dfne_weights_v1_0.pth")


### 4.2 Typical Evaluation Workflow

A typical evaluation workflow for using DFNE includes several steps:

1. Perform object detection to localize animals within images (needed for bounding box creation).
2. Analyze the cropped images using DFNE.
3. Perform post hoc analysis on DFNE model outputs.

In the minimal example, we use pre‐calculated bounding boxes (rather than generating them via object detection). Here, we will read in the image labels (with pre‐calculated bounding boxes) and save the image crops using the save_crops() function, which returns a data frame mapping the cropped image file names to the original files:photo_dir = "data/eval/images" # Path to the folder containing sample imagescrops_dir = "temporary_directory" # Path to a temp directory for image cropsimage_data = pd.read_csv("data/eval/metadata/eval_dataset_demo.csv")# Save image cropscropped_images = save_crops( photo_dir = photo_dir, save_dir = crops_dir, d = image_data)


Next, we can classify the images using the DFNE batch_image_classify() function, which returns predicted class and a confidence score for each cropped image. The softmax argument specifies whether to return confidence scores for each class, also known as “softmax” values, that sum to 1 across all classes.dfne_detections = classifier_model.batch_image_classify( data_path = crops_dir, # Path to the folder containing images softmax = True # Whether to include softmax values for each detection)


Finally, we can perform any post‐processing steps. Several utility functions give the ability to save classification metadata in either the COCO Camera Traps data format (Beery et al. 2019) or in a tabular format amenable for importing into an *AMMonitor* database (Clarfeld, Tang, et al. 2025). Here, we compare the predicted classes of cropped images with the original labels for each bounding box to assess the classification accuracy:# Determine whether each prediction is correcty_correct = dfne_detections['y_pred'] == image_data['taxon']print(f"Classification accuracy: {sum(y_correct)/len(y_correct)*100}%")


**FIGURE 3:**
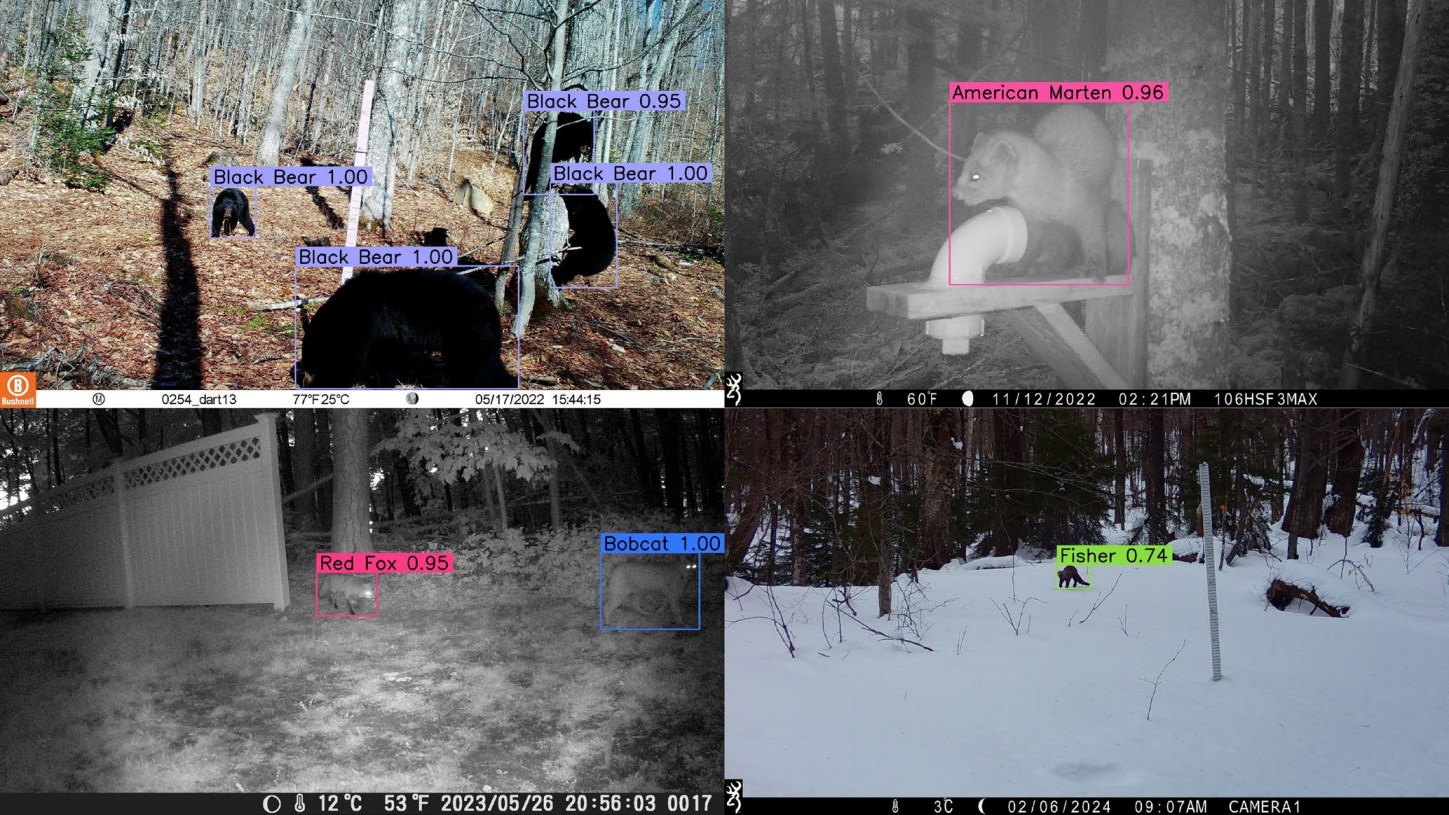
Annotated trail camera photos depicting bounding boxes around detected animals with the taxon labels and confidence scores for each box.

A full, working example of image analysis is included as a Jupyter Notebook (Kluyver et al. 2016) that comes with the source code (demo_eval.ipynb). Users should view this notebook for implementation details. A sample of images annotated with DFNE are included in Figure 3.

### 4.3 Training New Models

The repository also includes code for re‐training the DFNE model on new data with the ability to add, remove, or modify taxa that can be identified.

A typical training workflow includes:

1. Select the base model to re‐train and form a training dataset.
2. Perform object detection to localize animals within images.
3. Save cropped images (these will be your training images).
4. Evaluate a subset of training data with the base model to form a taxonomic mapping (using the map_taxa() function).
5. Initialize the base model based on the taxonomic mapping.
6. Re‐train the base model (using the train() function).

The Jupyter Notebook demo_train.ipynb contains code for re‐training the original DeepFaune model to classify American Marten vs. Fisher using a small training set of 50 images. The notebook gives users a working example to build from when training their own models.

### 4.4 Using DFNE With Pytorch‐Wildlife

The DFNE model has been fully integrated into Pytorch‐Wildlife (PW), a Python package that assimilates openly sourced models and tools for working with trail camera data into a single framework (Hernandez et al. 2024). PW integration allows users to leverage additional PW functionality for localizing and cropping animals (using several versions of MegaDetector), running the classifier, and post‐processing. Users who run DFNE via PW need only install Pytorch‐Wildlife with pip install PytorchWildlife(), which will include all other dependencies. The model weights will be downloaded automatically the first time the model is used via PW. A full example of the image classification pipeline using PW is included in an online demo (Hernandez et al. 2024).

## 5 Discussion

DeepFaune New England offers those in northeastern North America a regional model for automating the identification of 24 taxa. DFNE requires an object detection model to localize animals in an image and then predicts the taxon of each animal. Training images represent a wide variety of locations, seasons, camera models, and include both baited and non‐baited protocols, improving the generalizability to different camera trapping methodologies. The model achieved high (97%) accuracy when evaluating out‐of‐sample data. However, several caveats remain.

The data used to train DFNE contained noisy labels. Most of the images from the Northeast were labeled by trained interns but were not validated, so they may contain errors. Additionally, while we only used bounding boxes with confidence scores of at least 0.75 to build our training, validation, and testing sets for DFNE, there were likely false positives (i.e., bounding boxes that do not contain animals) included in the training data. We reviewed 500 randomly sampled training images and found five (1%) were mislabeled as having an animal when they were empty. Despite being trained with noisy labels, the model was able to learn effectively and achieve high performance. While DFNE was trained to classify false positive bounding boxes with the “no‐species” label, users may also opt to only classify predicted bounding boxes that have high confidence scores (e.g., > 0.75).

Although our model performed well when evaluated on out‐of‐sample images, further testing will reveal the generalizability of the model to new locations and data collection protocols. DFNE likely has utility outside of the northeast region, in areas where the covered taxa overlap significantly such as the mid‐Atlantic states, adjacent Canadian provinces, and the temperate forests of the Midwest. However, few trail camera images from semiaquatic habitats were available for training, so species such as American mink ( *Mustela vison* ), North American river otter ( *Lontra canadensis* ), common muskrat ( *Ondatra zibethicus* ), and American beaver ( *Castor canadensis* ) were not covered by the model. Species at the southern periphery of the region (e.g., cottontail (*Sylvilagus* sp.)) and along the north (e.g., Canada lynx ( *Lynx canadensis* )) were also excluded due to low sample size. These excluded taxa are likely to be misclassified (e.g., a mink may be misclassified as a fisher), so manual verification of predictions is encouraged. As additional training data become available, this model could be updated to provide more comprehensive species coverage.

DeepFaune New England exemplifies how open‐source software and openly licensed data can accelerate the development of high‐performance ML models. Data collection, curation, and labeling are often time‐consuming and expensive tasks, and the lack of adequate training data can limit the ability of ML practitioners to train new models. Over 75% of the images from our training set are publicly available through online repositories for future ML practitioners to use in training new models. Integration into open‐source frameworks such as Pytorch‐Wildlife makes the model more accessible and available to researchers who wish to integrate the animal detection, species classification, and post‐processing steps into a unified processing pipeline.

The development of DFNE was enabled by an ecosystem of open source tools and products, including the Pytorch programming framework, base model architecture, and DeepFaune model weights. Further, by integrating the model with Pytorch‐Wildlife, we can leverage the tools of that package while contributing to the open source community with this new, regional model, allowing for easy integration into camera trapping workflows. We hope that DFNE serves as both a useful tool for those working with trail camera data and as a foundation for expanding regional species classification model development across North America.

## Author Contributions

**Laurence A. Clarfeld:** conceptualization (lead), data curation (lead), formal analysis (lead), methodology (lead), software (lead), validation (lead), writing – original draft (lead), writing – review and editing (lead). **Katherina D. Gieder:** conceptualization (supporting), data curation (supporting), funding acquisition (lead), resources (lead), supervision (lead), writing – original draft (supporting), writing – review and editing (supporting). **Angela Fuller:** data curation (supporting), writing – review and editing (supporting). **Zhongqi Miao:** software (supporting), writing – review and editing (supporting). **Alexej P. K. Sirén:** data curation (supporting), writing – review and editing (supporting). **Shevenell M. Webb:** data curation (supporting), writing – review and editing (supporting). **Toni Lyn Morelli:** data curation (supporting), writing – review and editing (supporting). **Tammy L. Wilson:** data curation (supporting), writing – review and editing (supporting). **Jillian Kilborn:** data curation (supporting), writing – review and editing (supporting). **Catherine B. Callahan:** data curation (supporting), writing – review and editing (supporting). **Leighlan S. Prout:** data curation (supporting), writing – review and editing (supporting). **Rachel Cliche:** data curation (supporting), writing – review and editing (supporting). **Riley K. Patry:** data curation (supporting), writing – review and editing (supporting). **Christopher Bernier:** data curation (supporting), writing – review and editing (supporting). **Susan Staats:** data curation (supporting), writing – review and editing (supporting). **Scott Wixsom:** data curation (supporting), writing – review and editing (supporting). **Therese M. Donovan:** conceptualization (supporting), data curation (supporting), funding acquisition (equal), resources (equal), supervision (equal), writing – original draft (supporting), writing – review and editing (supporting).

## Conflicts of Interest

The authors declare no conflicts of interest.

## Figures and Tables

**FIGURE A1:**
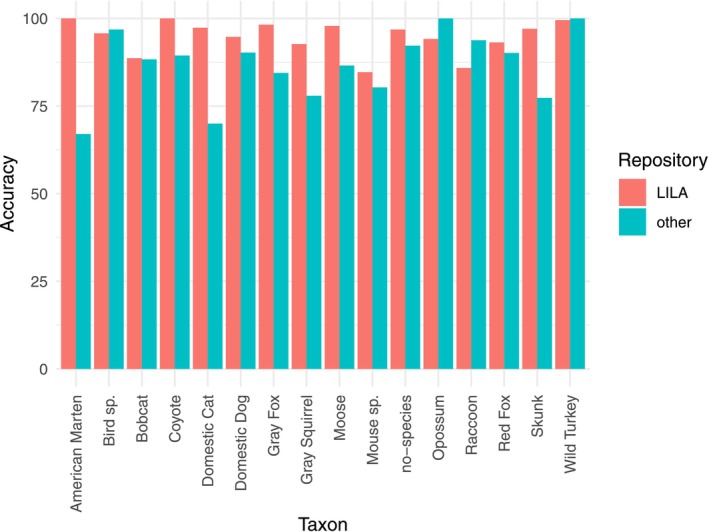
Accuracy of SpeciesNet, by taxon, for images that were part of a dataset from the LILA repository (Caltech Camera Traps, ENA24‐detection, and North American Camera Trap Images) vs. images from other datasets. Accuracy is calculated using the validation dataset and excludes taxa that were represented by images from either LILA or another dataset (but not both).

## Data Availability

The Deep Faune New England code and model weights have been officially released by USGS. The main code repository is at https://code.usgs.gov/vtcfwru/deepfaune‐new‐england (Clarfeld, Tracey, et al. 2025) and the model weights and training metadata are available on USGS ScienceBase at https://doi.org/10.5066/P1E7NDAF (Clarfeld, Gieder, et al. 2025).

## Appendix A

SpeciesNet is a Python package released in 2025 that provides an ensemble of models for classifying wildlife in trail camera imagery (Gadot et al. 2024). It is trained to classify over 2000 taxa from around the world. For our model comparison we extracted the logit scores from SpeciesNet predictions using the “target_species_txt” parameter, matching the 24 taxa classified by DFNE for a 1‐to‐1 mapping of classes between models. SpeciesNet includes an object detection tool for localizing animals, but we analyzed the same image crops that formed the DFNE validation set to ensure each model received the same input data.

SpeciesNet achieved 90% accuracy (−7% from DFNE) on the validation set. Precision and recall were well‐balanced and are presented, with F1 score, in Table A1. Despite high overall performance, some taxa showed poorer results, with Red Squirrel ( *Tamiasciurus hudsonicus* ), American Marten ( *Martes americana* ), and Snowshoe Hare ( *Lepus americanus* ) all obtaining under 70% accuracy. Over half (54%) of SpeciesNet misclassifications predicted “no‐species” when the cropped image contained an animal.

**TABLE A1:** Comparison of performance metrics, averaged across classes, between DeepFaune New England (DFNE) and SpeciesNet. Metrics include precision, recall, and F1 score (a harmonic average of precision and recall).

Metric	DFNE	SpeciesNet
Precision	0.97	0.92
Recall	0.97	0.89
F1	0.97	0.90

We also considered that SpeciesNet was trained using data from the LILA (Labeled Information Library of Alexandria) repository (Gadot et al. 2024), which may result in biased evaluation results where SpeciesNet was evaluated on a subset of its own training data. We compared classification accuracy for each taxon based on whether the validation data were from the LILA repository and found for 75% of taxa, SpeciesNet performed better on data from LILA, suggesting possible overfitting and that SpeciesNet accuracy on out‐of‐sample data may be lower than this comparison suggests (Figure A1).

The performance of DFNE demonstrates that regional models may allow for specialization in the identification of taxa particular to a specific region compared to global models. However, there are several important caveats to this model comparison. First, the DINOv2 ViT architecture used by DFNE is larger (~300 M vs. ~52 M parameters) and more computationally intensive (~507 G vs. ~24 G FLOPs) than SpeciesNet's EfficientNetV2‐M architecture (Tan and Le 2021; Oquab et al. 2023), making SpeciesNet more desirable on edge devices or when computational efficiency is desired. SpeciesNet can also classify many more taxa than DeepFaune New England, so it has utility in regions where the taxa present are not fully captured by DFNE.
